# Effect of Bamboo Flour Grafted Lactide on the Interfacial Compatibility of Polylactic Acid/Bamboo Flour Composites

**DOI:** 10.3390/polym9080323

**Published:** 2017-07-30

**Authors:** Xin-Yu Song, Meng Wang, Yun-Xuan Weng, Zhi-Gang Huang

**Affiliations:** 1 School of Materials and Mechanical Engineering, Beijing Technology& Business University, Beijing 100048, China; Songxy722@163.com (X.-Y.S.); dywewm@163.com (M.W.); huangzg@btbu.edu.cn (Z.-G.H.); 2 Beijing Key Laboratory of Quality Evaluation Technology for Hygiene and Safety of Plastics, Beijing Technology and Business University, Beijing 100048, China

**Keywords:** bamboo flour, lactide, graft, polylactic acid, compatibility

## Abstract

Bamboo flour (BF) was grafted onto lactide (LA) in the molten state using stannous octoate as a catalyst to form BF-*g*-LA. Then, polylactic acid (PLA) was blended with BF (PLA/BF, 85/15 wt %) to prepare PLA/BF/BF-*g*-LA composites using BF-*g*-LA as a compatibilizer. The grafting rate of BF was characterized using infrared testing and elemental analysis. To investigate the effect of BF-*g*-LA on the performance of PLA/BF/BF-*g*-LA composites, the phase morphology, thermal stability, and mechanical properties of the composites were characterized using scanning electron microscopy, thermogravimetric analysis, and universal material testing, respectively. The addition of BF-*g*-LA improved the interface compatibility between PLA and BF. When the BF-*g*-LA content was 2 phr, the tensile and impact strengths of PLA/BF/BF-*g*-LA composites were 55.3 MPa and 9.56 kJ/m^2^, representing 30% and 27% increases, respectively, relative to corresponding values for PLA/BF composites.

## 1. Introduction

The heavy use and unreasonable disposal of plastics has exacerbated environmental pollution in recent years. Thus, the development of environmentally sustainable replacements for traditional plastics is becoming a hot topic of today’s research and development [1,2]. Polylactic acid (PLA) is a biodegradable, biological base material made from renewable resources. Similar to traditional plastics, PLA can be processed with thermoplastic methods, and it has excellent physical properties such as high transparency, biocompatibility, and biodegradability; however, it is expensive to produce and it is not heat-resistant, which limits its rapid development [3,4,5]. Many researchers have investigated ways to reduce the cost of PLA production. For example, composites have been prepared by blending PLA with starch, wood fiber, bamboo fiber, cotton, linen, and other natural polymers or inorganic fillers. Lv et al. [6], Chuayjuljit et al. [7] and Liu et al. [8,9] blended wood powder with PLA, and Sajna et al. [10] blended banana fiber with PLA to prepare composite materials. Nezamzadeh et al. [11] prepared thermoplastic/starch composites (PLA/TPS) by melt blending PLA. Gunti et al. [12,13] prepared natural fiber reinforced biodegradable material with short jute and big elephant grass fibers blended with PLA. Jiang et al. [14] and Prasad et al. [15] investigated the mechanical and thermal properties of fiber reinforced PLA composites by treating the surface of sisal fibers with alkali and silica aerogels (SAG). Arjmandi et al. [16] enhanced PLA by blending it with cellulose nanowhiskers (CNWs) and montmorillonite (MMT), and then investigated the mechanical properties of the composites. Gu et al. [17] and Almeida et al. [18] observed the morphological and rheological behavior of PLA/CaCO_3_ composites, and Picard et al. [19] investigated the crystallinity and barrier properties of montmorillonite/PLA composites.

Bamboo flour (BF) is a renewable, low-cost, natural polymer material; therefore, it is often blended with plastic to prepare bamboo/plastic composite materials. Wang et al. [20] grafted GMA onto PLA to synthesize PLA-*g*-GMA using benzoyl peroxide (BPO) and tert-butyl peroxy benzoate (TBPB) as initiators, and the PLA-*g*-GMA was used as a compatibilizer for PLA/BF composites. It was determined that PLA-*g*-GMA improved the interface compatibility between PLA and BF. Kang et al. [21] prepared PLA/BF composites by blending BF and PLA with a silane coupling agent, maleic anhydride, and acrylic acid. Wang et al. [22] treated bamboo flour with a sodium silicate solution, and then blended it with PVC to prepare PVC/BF composites. Lu et al. [23] and Lee [24] prepared PLA/BF and PP/BF composites by blending BF with PLA and PP, respectively, after alkali treatment. Xu et al. [25] and Ochi [26] prepared PBS/acetylated-BF composites by blending polybutylene succinate (PBS) with acetylated bamboo fiber. Gupta [27] prepared epoxy resin/BF composites using bamboo fiber reinforced with epoxy resin.

In this study, BF-*g*-LA was synthesized by grafting BF onto LA in the molten state using stannous octoate as a catalyst. Using BF-*g*-LA as a compatibilizer, we prepared PLA/BF/BF-*g*-LA composites by blending polylactic acid (PLA) with BF (PLA/BF, 85/15 wt %) and investigated the effect of BF-*g*-LA on the performance of the composites. The use of BF-*g*-LA as a compatibilizer for PLA and BF melt blending has not been reported previously.

## 2. Experimental Procedure

### 2.1. Materials and Reagents

The following materials and reagents were used in this study: PLA (4032D, density: 1.24 g/cm^3^, melt index: 7 g/10 min (190 °C, 2.16 Kg), NatureWorks company (Minnetonka, MN, USA)); BF (80–120 mesh, Mujiang Weihua Spice Factory, Jiangmen, China); stannous octoate (pure grade, Aladdin Chemistry Co. Ltd., Shanghai, China); toluene (pure grade, Beijing Chemical Factory, Beijing, China); chloroform (pure grade, Beijing Chemical Factory, Beijing, China); LA (pure grade, Plac Company Shanghai Representative Office, Shanghai, China); and antioxidant (YD-1010, Beijing Dilong Chemical Co. Ltd., Beijing, China).

### 2.2. Principal Equipment Used

#### 2.2.1. Grafting Reaction

The following laboratory equipment was used in this procedure: a collective thermostatic heating magnetic stirrer (DF-101S, Bangxi Instrument Technology Co. Ltd., Shanghai, China); a horizontal constant speed electric stirrer (HD2004W, Shanghai Secretary Music Instrument Co. Ltd., Shanghai, China); a circulating aquatic multi-purpose vacuum pump (SHB-2IIIA, Linhai Yonghao Vacuum Equipment Co. Ltd., Zhejiang, China); a three-necked flask (1000 mL, Sichuan Shu Glass (Group) Co. Ltd., Sichuan, China); a beaker (500mL, Sichuan Shubo (Group) Co. Ltd., Sichuan, China); and glass rods (6 mm × 250 mm, Beijing Humanities Huatai Biotechnology Co. Ltd., Beijing, China).

#### 2.2.2. Blending

The following laboratory equipment was used in this procedure: an XSS-300 Torque Rheometer (E85-582, Shanghai Branch Chong Rubber Machinery Equipment Co. Ltd., Shanghai, China); a flat press (LP-S-50, Sweden LAB TECH, Boston, MA, USA); a microcomputer-controlled electronic universal testing machine, UTM (Chengde Jin Jian Detection Instrument Co. Ltd., Hebei, China); a cantilever beam impact testing machine, (XJUD-55, Chengde Jin Jian Detection Instrument Co. Ltd., Hebei, China); a thermogravimetric (TG)tester (Q5000IR, TA Co., New Castle, DE, USA); an infrared spectrometer (Nicolet iZ10, Thermo Scientific Co., Waltham, MA, USA); an elemental analyzer (vario EL, Elementar, Shanghai, China); a scanning electron microscope (TESCAN VEGA II, TESCAN s.r.o, Brno, Czech); a vacuum oven (DZG-6050, Shanghai Senxin Experimental Instrument Co. Ltd., Shanghai, China); and an electric air-blowing dryer (A101-E3, Shanghai Second Hardware Factory, Shanghai, China).

### 2.3. Sample Preparation

#### 2.3.1. Preparation of BF-*g*-LA

First, BF was dried in a vacuum oven at 80 °C for 12 h, and the appropriate amount of LA was dried in a vacuum oven at 40 °C for over 12 h.

**Figure 1:**
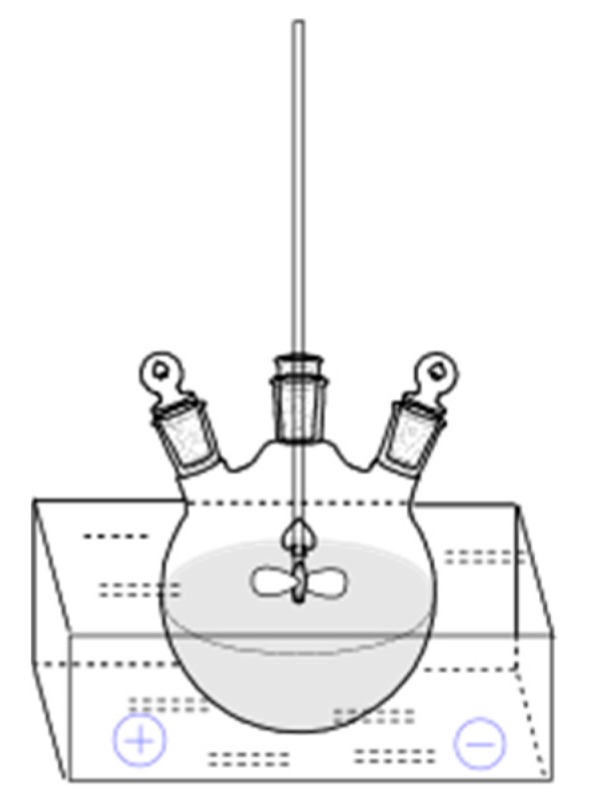
Experimental reaction device.

The dried BF and LA were poured into a three-necked flask, and the grafting reaction was initiated. The reaction device is shown in Figure 1, and the specific material mass ratio, reaction temperature, stirring speed, and reaction time are listed in Table 1.

Annotations: *T*_Melt_, LA melting temperature; *T*_swell_, BF swell temperature; *T*_reaction_, LA-BF reaction temperature; *v*_Melt_, LA agitation speed when melted; *v*_swell_, BF agitation speed when swelled; *v*_reaction_, LA–BF reaction stirring speed; *t*_melt_, time of LA melting; *t*_swelling_, time of BF swelling; *t*_reaction_, LA–BF reaction time.

The experimental steps for the BF–LA grafting reaction are as follows:

(1) LA melting stage: The LA was weighed and placed into a three-necked flask. An oil bath was gradually warmed to 100 °C, the stirrer was initiated, and the agitator gradually accelerated from a low speed to 150 r/min until all of the LA had melted (within 1 h).

(2) BF swelling stage: BF was added to the three-necked flask, and the contents were mixed evenly at a stirring speed of 180 r/min and a temperature of 100 °C for 5 h. During this process, BF was sufficiently swollen in the molten state of LA.

(3) LA and BF reaction phase: A distilled and dried toluene solution containing 2% stannous octoate (weight of LA) was added to the LA/BF solution. At the same time, the stirring speed was increased to 220 r/min and the temperature was elevated to 110 °C, initiating the chemical reaction between LA and BF. When the viscosity of the solution began to increase rapidly (about 1.5 h after the start of the reaction), stirring was stopped and the reaction proceeded for 6 h, after which heating was stopped.

(4) Washing the reaction product: After the reaction product cooled, a chloroform solution was added to the three-necked flask and heated to 60 °C from a low temperature. After the reaction product was completely dissolved, it was centrifuged. The above procedure was repeated four times to rinse off the untreated LA and free oligomers. The obtained BF-*g*-LA was dried in a vacuum at 60 °C for 8 h.

#### 2.3.2. Preparation of PLA/BF/BF-*g*-LA Composites

BF was dried for 12 h in a vacuum oven at 80 °C, and PLA was dried in a vacuum oven at 80 °C for 8 h.

The mixture formulation of the PLA/BF/BF-*g*-LA composite material is shown in Table 2.

According to Table 2, we weighed PLA and BF, mixed them evenly together, and transferred them to a mixer. The mixture was kneaded for 480 s at 180 °C and 40 rpm. The blended materials were then pressed into a 4-mm-thick flat plate at 190 °C by compression molding. The stretch spline conformed to GB/T1040-92 type I dumbbell specimens, and the impact spline conformed to GB/T1843-2008 non-gap specimens.

### 2.4. Performance Testing and Characterization

#### 2.4.1. Determination of BF-*g*-LA Content

(1) Elemental analysis

A 3-mg sample of BF-*g*-LA was tested with an elemental analyzer (CHN mode). The temperature in the oxidation tube was 1150 °C, and that in the reduction tube was 850 °C. Four sets of samples were tested, and the mean values were calculated.

(2) Infrared spectroscopy

The samples were blended with KBr powder and pressed into sheets. The sheets were scanned using an infrared spectrometer with a resolution of 4 cm^−1^ and a scanning frequency of 32 in the range of 400–4000 cm^−1^.

#### 2.4.2. Thermogravimetric Analysis of BF-*g*-LA Modified PLA/BF Composites

The sample was subjected to thermogravimetric analysis under a nitrogen atmosphere (40 mg/min) at a heating rate of 20 °C/min. The determination range was 50–600 °C.

#### 2.4.3. Testing of Mechanical Properties

The samples were placed in a laboratory environment for 24 h and subjected to tensile testing according to GB/T 1040.2-2006 (IDT ISO 527.2-2: 1993) with a universal testing machine at a tensile speed of 2 mm/min.

Non-notched impact tests were performed using a cantilever beam impact testing machine (1J hammer) according to GB/T 1843-2008 (IDT ISO 180-2000).

#### 2.4.4. Scanning Electron Microscopy

The fracture surfaces of samples were sprayed with gold and observed at 2000× magnification with an acceleration voltage of 10 kV.

## 3. Results and Discussion

### 3.1. Characterization of BF-g-LA

#### 3.1.1. Infrared Spectroscopy

**Figure 2:**
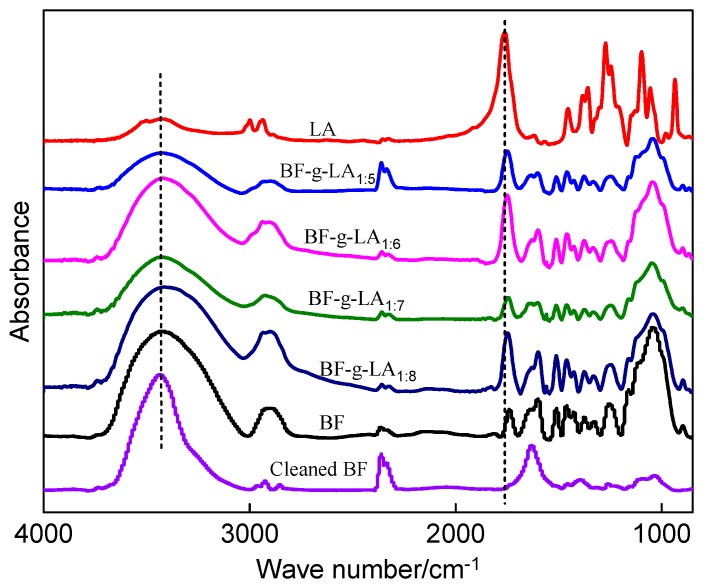
Infrared spectra of BF-*g*-LA.

The infrared spectra of BF-*g*-LA are shown in Figure 2, and the infrared absorption peaks for the main groups are listed in Table 3.

As shown in Figure 2 and Table 3, the carbonyl stretching vibration peak at 1740–1762 cm^−1^ disappeared after BF was rinsed with chloroform. The characteristic absorption peaks of the C–H bond at 1425 and 1460 cm^−1^ (due to C–H bending and stretching) and the benzene ring stretching vibration peak at 1330 cm^−1^ also disappeared. The disappearance of these peaks indicated that the structure of the BF was destroyed after rinsing with chloroform. However, these peaks still existed after BF was grafted onto LA, indicating that LA reacted with cellulose, hemicellulose, and lignin in the BF and the reaction product could not be rinsed with chloroform. In addition, the small molecular initiator, LA, free oligomer, and polymer were washed with chloroform to obtain high-purity BF-*g*-LA, so as to avoid holes formed by the small molecular initiator, LA, free oligomer cause poor compatibility between BF and PLA [28].

**Figure 3:**
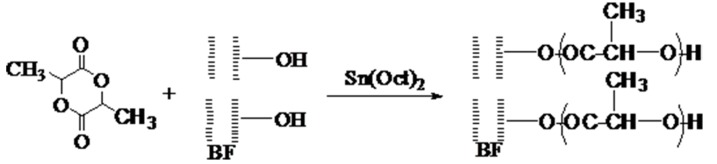
Reaction mechanism for Bamboo flour (BF) grafting lactide (LA).

A model describing the reaction of the LA–BF grafting reaction is shown in Figure 3.

The cell wall of BF fiber generally contains 40–50% cellulose, 20–30% hemicellulose, and 15–25% lignin in addition to small amounts of pectin, starch, pigment, inorganic materials, and other components. Cellulose is a linear homogeneous polymer formed by the polymerization of d-glucose via β-1,4-glycosidic bonds. Hemicellulose is a class of complex heterogeneous glycans consisting of pentose (xylose and arabinose), hexose (mannose, glucose, and galactose) and uronic acid. Lignin is a non-homogeneous amorphous polymer that contains three phenylpropane units (pineal alcohol, mustard alcohol, and coumarin).The benzene-propane units are linked by ether linkages (–O– and carbon-carbon bonds (–CC–).The hydroxyl groups in these components can be polymerized with lactide after the rings open, causing grafting of lactide onto BF.

The open ring of LA reacted with the hydroxyl groups on the surface of BF, and the resulting molecular chain was longer in the BF-*g*-LA product (Figure 3); therefore, the product was no longer soluble in chloroform.

After washing with chloroform, the absorption peak of BF-*g*-LA was larger than that of BF, and the absorption peak of the carbonyl group at 1740 cm^−1^ shifted. The reason for these changes is that the stretching vibration peaks caused by carbonyl in BF-*g*-LA shifted between 1740 and 1762 cm^−1^ when LA was grafted onto the BF molecular chain [20].

The hydroxyl stretching vibration peak occurred at 3500 cm^−1^. The polarity of BF was strong owing to hydrogen bonding of the hydroxyl group. After BF was grafted, the molecular chain linked to the hydroxyl group lengthened and the polarity decreased. Subsequently, the strength of the hydrogen bonds decreased [29], and the intensity of the hydroxyl-induced stretching vibration peak was reduced.

#### 3.1.2. Elemental Analysis

The carbon content in LA, BF, and BF-*g*-LA was determined using elemental analysis (Table 4).

According to Table 4, the C content of pure BF, cleaned BF (with chloroform), and LA was 49.83%, 49.26%, and 48.80%, respectively. The C content of the grafted products BF-*g*-LA_1:5_, BF-*g*-LA_1:6_, BF-*g*-LA_1:7_, and BF-*g*-LA_1:8_ was 48.86%, 48.91%, 48.97%, and 49.02%, respectively. These data indicate that LA was successfully grafted onto the BF molecular chain, which is consistent with results of the infrared spectroscopy analysis.

The content of LA in the grafted copolymer was calculated with Equation (1)

(1) $$w_{2}=\frac{x_{3}\;-\;x_{1}w_{1}}{x_{2}}$$

where *x*_1_ is the carbon content of BF (wt %); *w*_1_ is the mass fraction of BF (wt %); *x*_2_ is the carbon content of LA (wt %); *w*_2_ is the mass fraction of LA (wt %); and *x*_3_ is the carbon content of the graft product (wt %) [20].

Based on the data in Table 4 and Equation (1), the content of LA in BF-*g*-LA_1:5_, BF-*g*-LA_1:6_, BF-*g*-LA_1:7_, and BF-*g*-LA_1:8_ was 94.17 ± 8.7%, 89.32 ± 12.6%, 83.50 ± 6%, and 78.64 ± 7.3%. It can be seen from the above analysis that the BF grafting rate was highest in BF-*g*-LA_1:5_. Therefore, BF-*g*-LA_1:5_ was used as a compatibilizer in subsequent experiments to study its compatibility in PLA and BF melt blending.

### 3.2. Effect of BF-g-LA on the Thermal Properties of Composites

**Figure 4:**
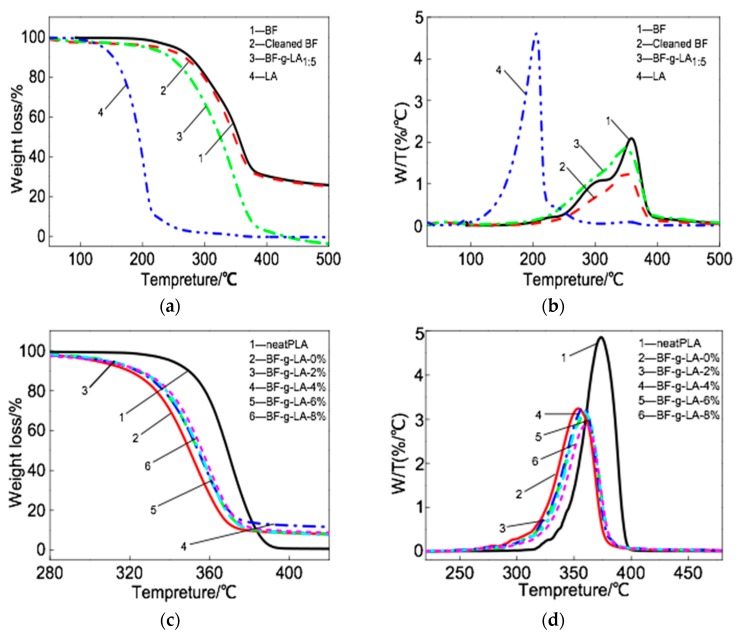
Thermal weightlessness curve for BF-*g*-LA and PLA/ BF/B-*g*-LA composites (**a**) TG curve for BF-*g*-LA; (**b**) DTG curve for BF-*g*-LA; (**c**) TG curves for PLA/BF/BF-*g*-LA composites; (**d**) DTG curve for PLA/BF/BF-*g*-LA composites.

The thermogravimetric curves (TG, DTG) of the BF-*g*-LA and PLA/BF/BF-*g*-LA composites are shown in Figure 4. The thermogravimetric temperature of BF-*g*-LA is shown in Table 5, and the thermogravimetric temperature of PLA/BF/BF-*g*-LA composites is shown in Table 6.

As seen in Figure 4a,b and Table 5, the 5% weight-loss temperature of BF was 251 °C, and the maximum decomposition rate temperature was 358 °C. The 5% weight-loss temperature of LA was 136 °C, and the maximum decomposition rate temperature was 205 °C. The 5% weight-loss and maximum decomposition rate temperatures of BF-*g*-LA_1:5_ were between the corresponding values for BF and LA because the presence of LA in the grafted product reduced the thermal decomposition temperature of BF-*g*-LA_1:5_. After washing with chloroform, hemicellulose and lignin in the BF were partly removed, and the weight loss temperature decreased.

The 5% weight-loss temperature of the PLA/BF composite without BF-*g*-LA was 302 °C and the maximum decomposition rate temperature was 353 °C (Figure 4c,d and Table 6). After adding 2, 4, 6, and 8 phr BF-*g*-LA, the 5% weight-loss temperature of the composites was 313, 311, 312, and 309 °C and the maximum decomposition rate temperature was 362, 361, 362, and 364 °C, respectively. Thus, the 5% weight-loss temperature and the maximum decomposition rate temperature for PLA/BF/BF-*g*-LA composites improved after the addition of BF-*g*-LA. When two phr BF-*g*-LA was added, the 5% weight-loss temperature of the composite was 313 °C and the maximum decomposition rate temperaturewas 362 °C (9 and 11 °C higher than that of PLA/BF composites without BF-*g*-LA, respectively).

The above analysis shows that the addition of BF-*g*-LA improved the compatibility between PLA and BF, and reinforced the thermal stability of PLA/BF composites.

### 3.3. Effect of BF-g-LA on the Mechanical Properties of Composites

**Figure 5:**
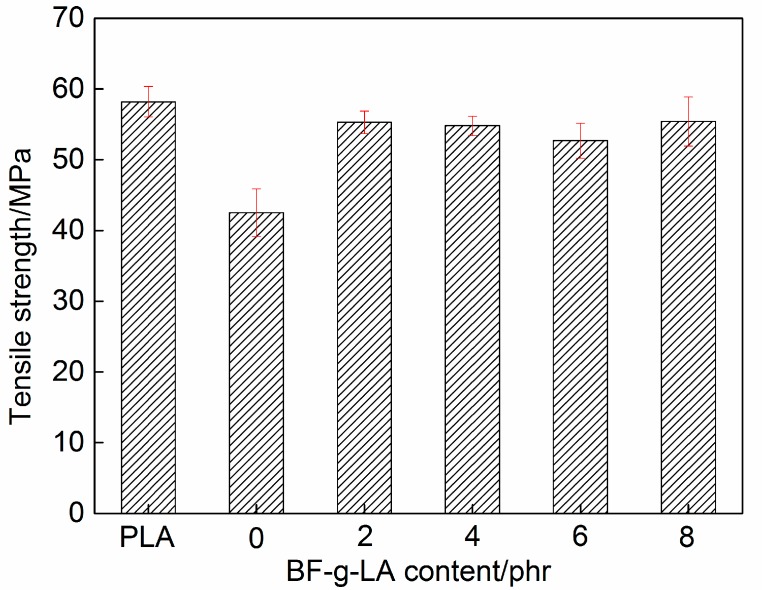
Tensile strength of PLA/BF/BF-*g*-LA composites.

**Figure 6:**
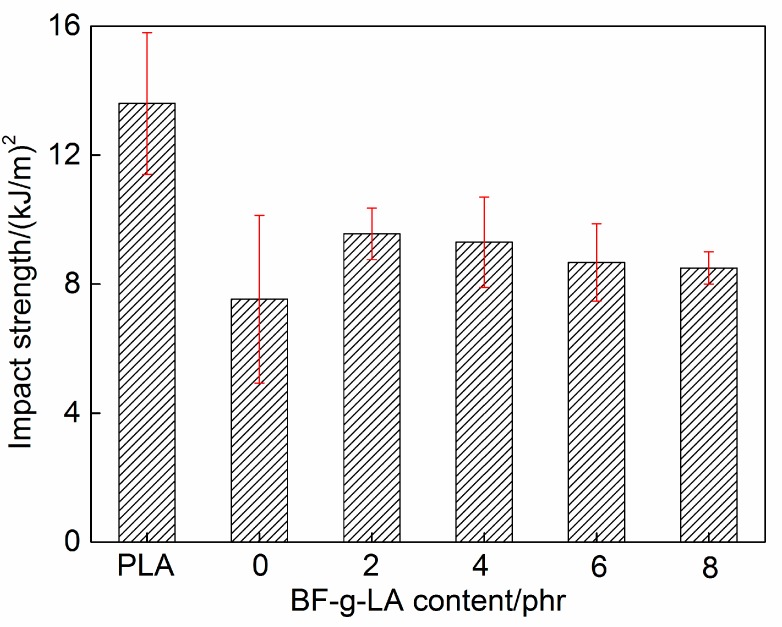
Impact strength of PLA/BF/BF-*g*-LA composites.

The measured tensile and impact strengths of PLA/BF/BF-*g*-LA composites are shown in Figure 5 and Figure 6, respectively.

The tensile strength of PLA/BF composites without BF-*g*-LA was 42.5 MPa, and the tensile strength of composites after addition of 2, 4, 6, and 8 phr BF-*g*-LA was 55.3, 54.8, 52.7, and 55.4 MPa, respectively (Figure 5). When the content of BF-*g*-LA was 8 phr, the tensile strength of the composite was 55.4 MPa, which is 30% higher than that of unmodified composites.

The impact strength of PLA/BF composites without BF-*g*-LA was 7.5 kJ/m^2^, and the impact strength of composites after addition of 2, 4, 6, and 8 phr BF-*g*-LA was 9.6 kJ/m^2^, 9.3 kJ/m^2^, 8.7 kJ/m^2^, and 8.5 kJ/m^2^, respectively (Figure 6). The impact strength of composites reached a maximum value of 9.6 kJ/m^2^ after addition of 2 phr BF-*g*-LA; this value was 27% higher than that of unmodified composites.

The tensile and impact strengths of PLA/BF composites were lower than corresponding values for pure PLA because the cellulose, hemicellulose, and lignin components of BF, contain large amounts of polar hydroxyl and phenolic hydroxyl groups (Figure 5 and Figure 6). The interaction between BF and hydrophobic surface PLA interface was weak. Therefore, stress could not be transmitted effectively along the interface. As a result, the tensile and impact strengths of the composites were low and the mechanical properties of the composites decreased [30,31]. However, after adding BF-*g*-LA, the structure of LA in BF-*g*-LA improved the compatibility between BF and PLA, causing the tensile and impact strengths to increase.

### 3.4. Effect of BF-g-LA on the Morphology of the Fracture Surface of the Composites

**Figure 7:**
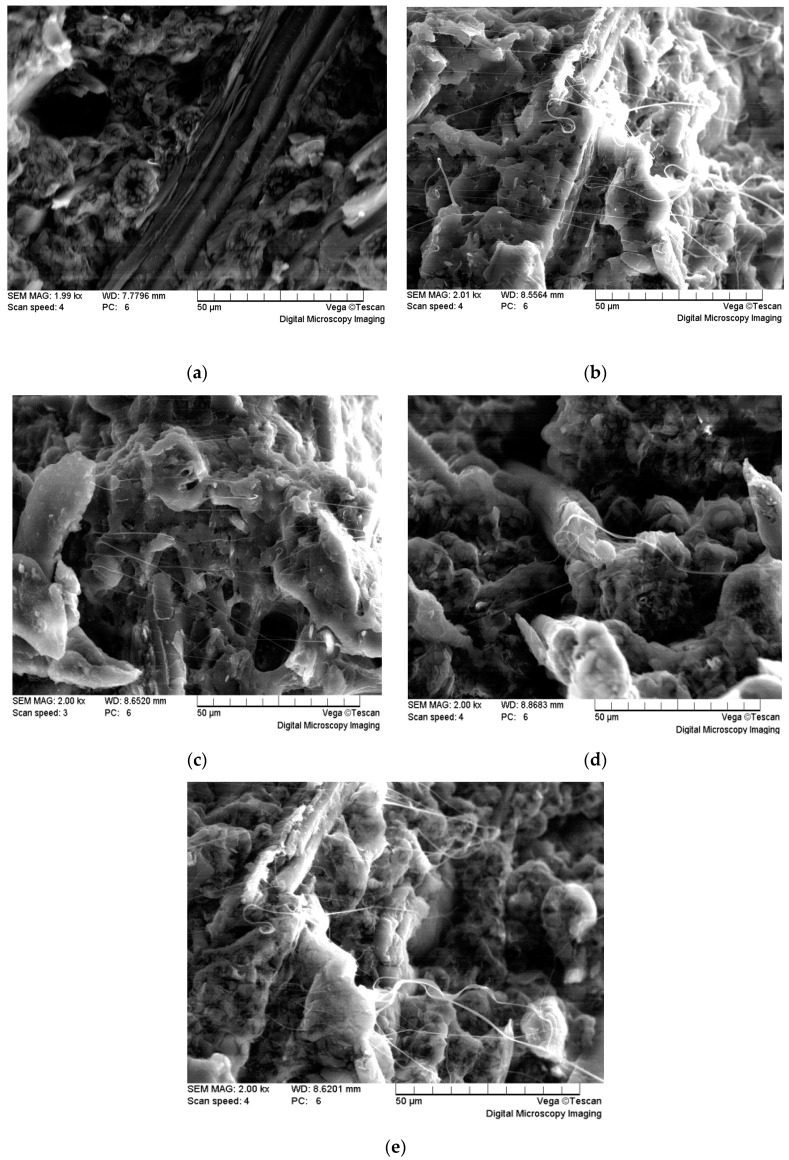
Scanning electron microscopy images (×2000) of PLA/ BF/BF-*g*-LA impact surfaces (**a**) BF-*g*-LA, 0 phr; (**b**) BF-*g*-LA, 2 phr; (**c**) BF-*g*-LA, 4 phr; (**d**) BF-*g*-LA, 6 phr; (**e**) BF-*g*-LA, 8 phr.

The morphologies of the impact fracture surfaces of PLA/BF/BF-*g*-LA composites are shown in Figure 7. The scanning electron microscopy image of the composite with no compatibilizer is shown in Figure 7a; composite images after the addition of BF-*g*-LA are shown in Figure 7b–e.

As shown in Figure 7a, without the addition of BF-*g*-LA, a clear phase separation occurred between BF and PLA after melt blending, indicating poor compatibility of the unmodified PLA/BF (85/15 wt %).

The compatibility between BF and PLA improved after the addition of BF-*g*-LA, and the interface of the matrix was tightly connected (Figure 7b–e). BF was dispersed and tightly enveloped in the PLA matrix. BF-*g*-LA acted as a reactive compatibilizer when the open ring in LA reacted with the hydroxyl group on the surface of BF, thereby forming filamentous structures and enhancing the compatibility between BF and PLA. As a result, the two phases were closely connected, forming a relatively stable and strong interface layer. After the impact, BF first bore stress for separation, but because of the strong force between the two phases, the fiber was accompanied by the PLA matrix resin during separation, resulting in adhesion of the drawing phenomenon [32].

## 4. Conclusions

(1) Stannous octoate can initiate BF grafting onto LA to obtain BF-*g*-LA. The LA content in BF-*g*-LA_1:5_, BF-*g*-LA_1:6_, BF-*g*-LA_1:7_, and BF-*g*-LA1 was 94.17%, 89.32%, 83.50%, 78.64%, respectively.

(2) The addition of BF-*g*-LA during the preparation of PLA/BF/BF-*g*-LA composites resulted in the formation of a relatively strong interfacial layer. The tensile strength, impact strength, and thermal decomposition temperature increased. The addition of BF-*g*-LA effectively improved the compatibility between PLA and BF. The tensile and impact strengths of PLA/BF/BF-*g*-LA composites (addition of 2 phr BF-*g*-LA) were 55.3 MPa and 9.6 kJ/m^2^, respectively, reflecting 30% and 27% increases compared with values for composites without BF-*g*-LA addition (42.5 MPa and 7.5 kJ/m^2^, respectively)

## Figures and Tables

**Table 1:** Experimental reaction conditions.

Grafting Product Abbreviation	BF/LAMmass Ratio (W_BF_/W_LA_)	Reaction Temperature (T/°C)			Stirring Speed (v/r·min^−1^)			Reaction Time (t/h)		
		*T*_Melt_	*T*_swell_	*T*_reaction_	*v*_melt_	*v*_swell_	*v*_reaction_	*t*_melt_	*t*_swelling_	*t*_reaction_
BF-*g*-LA_1:5_	1:5	100	100	110	150	180	220	1	5	6
BF-*g*-LA_1:6_	1:6	100	100	110	150	180	220	1	5	6
BF-*g*-LA_1:7_	1:7	100	100	110	150	180	220	1	5	6
BF-*g*-LA_1:8_	1:8	100	100	110	150	180	220	1	5	6

**Table 2:** Mixture formulation (wt %).

PLA/wt %	BF/wt %	BF-*g*-LA/phr	Antioxidant/phr
85	15	0	1
85	15	2	1
85	15	4	1
85	15	6	1
85	15	8	1

**Table 3:** Infrared absorption peaks of the main groups.

Wave Number/cm^−1^	Group
3500	Hydroxyl O–Hstretching vibration
1740–1762	Carbonyl C=O stretching vibration
1425, 1460	C–H bending, stretching vibration
1330	Benzene ring stretching vibration

**Table 4:** Carbon content of LA, BF, and BF-*g*-LA determined by elemental analysis.

Concentration	LA	BF	Cleaned BF by Chloroform	BF-*g*-LA_1:5_	BF-*g*-LA_1:6_	BF-*g*-LA_1:7_	BF-*g*-LA_1:8_
C wt %	48.80 ± 0.08	49.83 ± 0.26	49.26 ± 0.18	48.86 ± 0.09	48.91 ± 0.13	48.97 ± 0.06	49.02 ± 0.04

**Table 5:** Thermogravimetric temperature of BF-*g*-LA.

Samples	5% Weight-Loss Temperature/°C	DTG Peak Temperature/°C
BF	251	358
Cleaned BF by chloroform	224	349
BF-*g*-LA_1:5_	205	346
LA	136	205

**Table 6:** Thermogravimetric temperature of PLA/BF/BF-*g*-LA composites.

Samples	5% Weight-Loss Temperature/°C	DTG Peak Temperature/°C
neat PLA	341	373
PLA/BF/BF-*g*-LA-0phr	302	353
PLA/BF/BF-*g*-LA-2phr	313	362
PLA/BF/BF-*g*-LA-4phr	311	361
PLA/BF/BF-*g*-LA-6phr	312	362
PLA/BF/BF-*g*-LA-8phr	309	364
